# Knee Biomechanics During Cutting Maneuvers and Secondary ACL Injury Risk: A Prospective Cohort Study of Knee Biomechanics in 756 Female Elite Handball and Soccer Players

**DOI:** 10.1177/03635465241234255

**Published:** 2024-03-08

**Authors:** Lasse Mausehund, Tron Krosshaug

**Affiliations:** † Oslo Sports Trauma Research Center, Department of Sports Medicine, Norwegian School of Sport Sciences, Oslo, Norway; Investigation performed at the Norwegian School of Sport Sciences, Oslo, Norway

**Keywords:** anterior cruciate ligament, reinjury, reconstruction, kinematics, kinetics, football, return to sport

## Abstract

**Background:**

An athlete who returns to sport after an anterior cruciate ligament (ACL) injury has a substantially high risk of sustaining a new secondary ACL injury. Because ACL injuries most frequently occur during cutting maneuvers, such movements should be at the center of research attention.

**Purpose:**

To investigate whether knee biomechanical parameters during side-step cutting maneuvers differ between female elite athletes with and without a history of ACL injury and to evaluate whether such parameters are associated with future secondary ACL injury.

**Study Design:**

Cohort study; Level of evidence, 2.

**Methods:**

A total of 756 female elite handball and soccer players, of whom 76 had a history of ACL injury, performed a sport-specific cutting task while 3-dimensional kinematics and kinetics were measured. ACL injuries were registered prospectively over an 8-year follow-up period. Seven knee-specific biomechanical variables were the basis for all analyses. Two-way analyses of variance were applied to assess group differences, whereas logistic regression models served to evaluate associations between the knee-specific variables and future secondary ACL injury.

**Results:**

When players with a previous ACL injury performed the cutting maneuver with their ipsilateral leg, they exhibited lower knee abduction angles (mean difference [MD], 1.4°-1.5°; 95% CI, 0.2°-2.9°), lower peak knee flexion moments (MD, 0.33 N·m/kg^-1^; 95% CI, 0.18-0.48 N·m/kg^-1^), lower peak knee abduction moments (MD, 0.27 N·m/kg^-1^; 95% CI, 0.12-0.41 N·m/kg^-1^), and lower peak knee internal rotation moments (MD, 0.06 N·m/kg^-1^; 95% CI, 0.01-0.12 N·m/kg^-1^) compared with injury-free players. When players performed the cut with their contralateral leg, no differences were evident (*P* < .05). None of the 7 knee-specific biomechanical variables was associated with future secondary ACL injury in players with an ACL injury history (*P* < .05).

**Conclusion:**

Approximately 4 years after ACL injury, female elite team-ball athletes still unloaded their ipsilateral knee during cutting maneuvers, yet contralateral knee loading was similar to that of injury-free players. Knee biomechanical characteristics were not associated with future secondary ACL injury.

One of the most serious consequences of a primary anterior cruciate ligament (ACL) injury is the substantially high risk of sustaining yet another ACL injury, in both the ipsilateral and the contralateral knee. In a meta-analysis from 2016, Wiggins et al^53^ concluded that an athlete who returns to play after an ACL injury has a 20% chance of sustaining a new ACL injury, with the contralateral knee being slightly more susceptible than the ipsilateral knee. This can make a young athlete who returns to sport after ACL reconstruction 6 times more likely to sustain an ACL injury than his or her uninjured counterpart (ACL injury rates of 1.39 vs 0.24 per 1000 athlete-exposures, respectively).^44^ Interestingly, the high risk of sustaining a secondary ACL injury seems to be independent of sex,^42^ implying that the risk factors for a primary and secondary ACL injury might differ. The consequences of a secondary ACL injury are even worse than those of a primary ACL injury, irrespective of whether the new injury affects the ipsilateral or contralateral knee: The rate of return to preinjury level of sport is lower,^9,15^ the subjective^9,14^ and objective^14^ knee function is diminished, the health-related quality of life is decreased,^12^ and, in case of an ipsilateral reinjury, more radiographic evidence of osteoarthritis is present^14^ compared with after primary unilateral ACL injury.

To prevent secondary ACL injuries, a logical first step is to assess why players with a previous ACL injury are at elevated risk of sustaining a new ACL injury.^2^ The risk of sustaining a secondary ACL injury is multifactorial,^5,6^ with adverse movement biomechanics likely being an important intrinsic risk factor.^18^ Identifying such biomechanical risk factors, which are modifiable through training, can help us to develop successful, time-efficient, and customized prevention programs. Risk factors and hence effective prevention programs are likely to differ slightly between primary and secondary ACL injury, warranting distinct investigations. Because the biomechanical characteristics of movement, even during simple tasks such as walking,^17^ running,^41^ or stair negotiation,^16^ are altered for years after an ACL injury, it seems likely that biomechanics may play a particularly important role in the injury etiology of secondary ACL injuries. So far, we have limited knowledge on biomechanical risk factors for secondary ACL injury in female athletes. To the best of our knowledge, only 5 prospective studies^22,23,28,45,48^ have investigated associations between biomechanical variables and future secondary ACL injury. Two of those studies^22,23^ were exclusively performed in male athletes. The remaining 3 studies, which tested participants in either a double-leg vertical drop jump^28,45^ or a single-leg landing task,^48^ showed divergent results. Because noncontact ACL injuries in handball^36,37^ and soccer^10,13^ most frequently occur during cutting maneuvers, biomechanical analyses of such movements are of major importance and might be the key to successful injury prevention. So far, no prospective studies have assessed biomechanical risk factors during side-step cutting maneuvers in female athletes. Further, only 3 cross-sectional studies^30,47,51^ have compared biomechanical variables during cutting maneuvers between female athletes with and without a previous ACL injury. These studies had small sample sizes, and the findings were conflicting. Studies with larger sample sizes are required to provide clarity, as are studies assessing whether those differences are present in elite athletes.

To assess potential risk factors for secondary ACL injury in female elite athletes who play team-ball sports, this study had 2 aims. The first aim was to use a cross-sectional design to assess whether knee biomechanical parameters during side-step cutting maneuvers differ between players with and without a history of previous ACL injury. Also, we wanted to compare players who went on to sustain a secondary ACL injury with 3 groups: injury-free players, players with a primary ACL injury only, and players who went on to sustain a primary ACL injury. The second aim was to use a prospective cohort design to determine whether knee biomechanical parameters during side-step cutting maneuvers are associated with an increased risk of future secondary ACL injury.

## Methods

### Study Design and Participants

To gain a better understanding of the causes of secondary ACL injury, we combined a prospective cohort design with a cross-sectional design. This investigation is part of a large prospective cohort study aimed at identifying risk factors for noncontact ACL injuries among female elite handball and soccer players.^28,39,46,52^ The results of the primary a priori hypothesis have been reported previously.^28^ Secondary analyses should be considered exploratory but valuable to better understand the multifactorial cause of ACL injuries.

**Figure 1.**
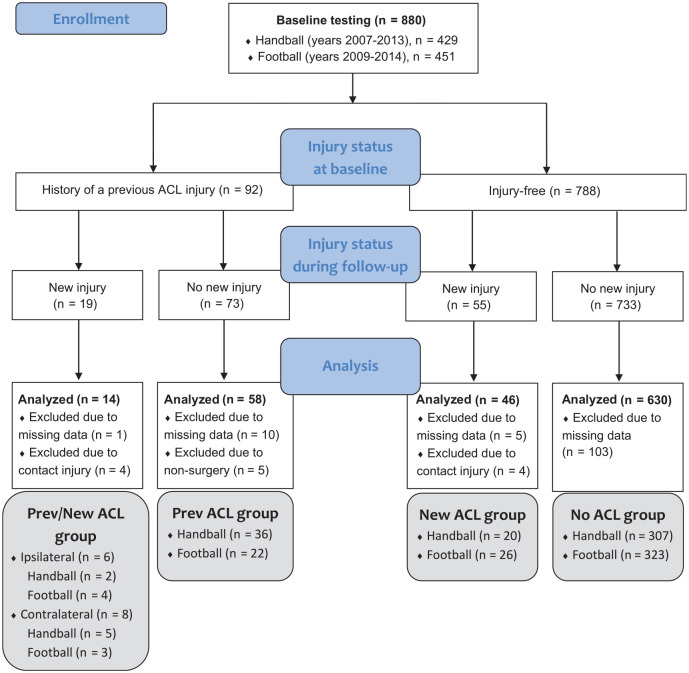
Flow diagram of the tested participants, including their injury status at baseline and follow-up and the number of analyzed participants in each group. All groups were included in the cross-sectional part of this study, whereas only the Prev/New ACL group and the Prev ACL group were included in the prospective part. ACL, anterior cruciate ligament; Prev, previous.

All data were collected over an 8-year period, starting in 2007, when all teams in the Norwegian female handball premier league were invited to participate in a comprehensive preseason baseline testing. To be included, players were expected to play in the premier league during the upcoming season and were required to have a first-team contract. Between 2008 and 2013, players from new teams joining the premier league and new players from already included teams were enrolled in the same preseason baseline screening. Between 2009 and 2014, female soccer players from the premier league were also included in the study, based on the same inclusion criteria. Almost 90% of the eligible players in the handball and soccer premier leagues were enrolled in this study. In total, 880 athletes, including 451 soccer players and 429 handball players, were tested. Of the players, 14% had to be excluded from the current study because of missing kinematic or kinetic data in the cutting task, which was caused by technical problems or illness or injury, or because they did not undergo ACL reconstruction surgery after a previous ACL injury (n = 5). Thus, the final sample consisted of 756 players (age, 20.8 ± 4.0 years; body mass, 66.2 ± 7.7 kg; height, 169.6 ± 6.3 cm), including 680 injury-free players, including the 4 players with contact injury and 76 players with a history of a previous ACL injury, including the 4 players with contact injury (Figure 1). The players with a previous ACL injury had sustained their injury a mean 3.6 ± 2.5 years previously.

The study was approved by the Regional Committee for Medical Research Ethics, the South-Eastern Norway Regional Health Authority, and the Norwegian Social Science Data Services. All participants provided written informed consent before inclusion, including parental consent for players aged <18 years. The study conformed to the latest revision of the Declaration of Helsinki.

### Data Collection and Test Procedures

All players attended 1 day of comprehensive baseline testing during the preseason, including a vertical drop jump task, a cutting task, and a range of neuromuscular, mobility, clinical, and anthropometric measurements. We obtained information about player demographic characteristics, playing experience, and history of previous ACL injuries through a detailed questionnaire.

**Figure 2.**
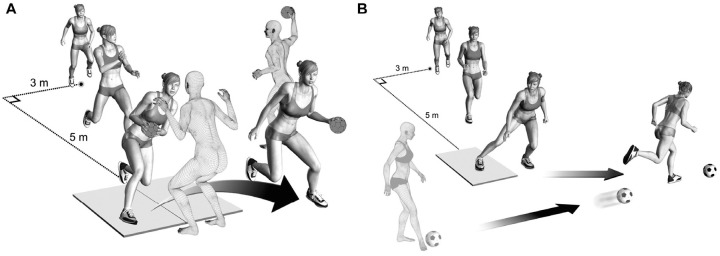
Cutting task illustration for (A) the handball-specific cut and (B) the soccer-specific cut. (Reprinted with permission from Mok KM, Bahr R, Krosshaug T. Reliability of lower limb biomechanics in two sport-specific sidestep cutting tasks. *Sports Biomech*. 2018;17(2):157-167. 2018, Taylor & Francis Ltd.)

The basis for this study was a biomechanical analysis of sport-specific cutting tasks using 3-dimensional motion capture. The players wore their own indoor shoes, shorts, and a sports bra. A total of 35 reflective markers were attached over uniquely defined marker positions.^27^ After a static, standing calibration trial and a standardized warm-up procedure, players commenced with the cutting task. They were instructed to execute the cutting maneuver as they would in a real match, with high intensity and effort. The players accelerated for 6 meters and arrived with matchlike, self-selected approach speeds at an angle of approximately 30° to the long axis of the runway (Figure 2). The cutting angle was not predetermined. For the handball-specific cut, the player received a lateral pass from a teammate right before performing a matchlike cutting maneuver to fake and pass a human static defender (Figure 2A). For the soccer-specific cut, the player received a pass forcing her to perform a sharp side-step cutting maneuver (Figure 2B). The details of the cutting test procedure were described previously.^35^ At least 5 successful trials with maximal matchlike effort were recorded for each leg, the first 3 of which were selected for analyses. If 1 or more markers were obscured during parts of the cutting movement or if the force platform was partially missed, we considered trials 4 and 5 as viable alternatives.

After baseline testing, all complete ACL ruptures were registered prospectively through May 2015, primarily through regular contact with the manager, coach, or medical staff of the participating teams. In case an acute knee injury was reported, we contacted the injured player directly to obtain medical data and a description of the injury situation. The injury mechanism was self-categorized as contact, indirect contact, or noncontact. All complete ACL ruptures were verified by magnetic resonance imaging and/or arthroscopy.

### Measurements and Data Processing

All measurements were synchronously collected through a 16-bit analog-to-digital conversion board (USB-2533; Measurement Computing Corporation), integrated to Qualisys Track Manager (Version 2.8; Qualisys AB) and further processed and analyzed in Matlab (Version 2011; MathWorks Inc). Between 2007 and 2012, the 3-dimensional kinematic data were recorded with an 8-camera motion capture system (ProReflex; Qualisys AB) sampling at 240 Hz. Beginning in 2012, an upgraded 16-camera system with a sample frequency of 480 Hz was used (Oqus 4; Qualisys AB). Ground-reaction forces and center of pressure were recorded using 2 force platforms (AMTI LG6-4-1) collecting data at 960 Hz.

Kinematic and kinetic data were filtered and interpolated using the same Woltring spline with a 15-Hz cutoff frequency to minimize effect artifacts.^27^ For each cut, the ground contact phase was defined as the period in which the unfiltered vertical ground-reaction forces exceeded 20 N. The position of the hip joint center was estimated using the method proposed by Bell et al,^3^ and the positions of the knee and ankle joint centers were estimated to be halfway between the epicondyle and malleoli markers, respectively.^28^ Hip and knee joint angles were calculated using the joint coordinate system convention, and joint moments were determined using common inverse dynamics calculations and reported as external joint moments.^28^*Approach speed* was defined as the velocity of the center of mass at initial contact (IC) and *cutting angle* as the angle between the velocity vector of the center of mass 42 ms before IC and 42 ms after toe-off.^26^

To reduce the risk of type I errors, we limited our analyses to the following 7 knee-related biomechanical variables measured during the ground contact phase of the cutting task: knee flexion angle at IC, peak knee flexion angle, knee abduction angle at IC, peak knee abduction angle, peak knee flexion moment, peak knee abduction moment, and peak knee internal rotation moment. For each leg, the mean of the 3 cutting trials was used as the basis for all analyses. Mok et al^35^ assessed the test-retest reliability of these variables during a cutting maneuver and demonstrated good to excellent within-session reliability and fair to good between-session reliability: knee flexion angle at IC (mean within-session intraclass correlation coefficient, 0.90; mean between-session intraclass correlation coefficient, 0.68), peak knee flexion angle (0.75, 0.63), knee abduction angle at IC (0.92, 0.55), peak knee abduction angle (0.95, 0.64), peak knee flexion moment (0.94, 0.80), peak knee abduction moment (0.90, 0.72), and peak knee internal rotation moment (0.95, 0.74). Mok et al concluded that adequate reliability could be attained from 3 trials. In addition to the 7 primary knee-specific variables mentioned above, we analyzed side-to-side differences for these variables where the asymmetry magnitude between legs was calculated with the root mean square difference between left and right leg.^25^ We chose to focus only on knee-specific variables in this study because they are more closely related to ACL loading than distant variables like trunk, hip, or ankle biomechanics. If variables such as lateral trunk flexion or foot rotation truly would be risk factors for ACL injury, their effect had to be mediated through the knee biomechanical variables that we analyzed in this study.

### Statistical Analysis

Statistical analyses were performed in IBM SPSS Statistics (Version 24; IBM Corporation).

For the cross-sectional part of this study, we performed 2 separate analyses. In the first analysis, we assessed differences between both the ipsilateral and the contralateral leg of players with an ACL injury history and a randomly selected leg of injury-free players.^39^ Because players with a previous ACL injury have an increased risk of a new injury in both the ipsilateral and the contralateral leg,^53^ we chose to assess each leg individually. In the second analysis, we compared players with a previous ACL injury who went on to sustain a new, secondary ACL injury (Prev/New ACL group) with the remaining 3 groups: that is, players with a previous ACL injury only (Prev ACL group) and players without a previous ACL injury who did (New ACL group) or did not (No ACL group) sustain a new, primary ACL injury during follow-up (Figure 1). Because previous research suggests differences in risk factors for ipsilateral reinjury and contralateral injury,^5,6,22,23^ we performed separate analyses for the ipsilateral knee and ipsilateral reinjury as well as for the contralateral knee and contralateral injury. Hence, in one analysis, the Prev/New ACL group included only players who went on to sustain a new ipsilateral reinjury, and for the Prev ACL group, we selected the ipsilateral leg. In the other analysis, the Prev/New ACL group included only players who went on to sustain a new contralateral injury, and for the Prev ACL group, we selected the contralateral leg. In both analyses, we used the leg that went on to sustain the new injury as the unit of analysis for the New ACL group, and we used a randomly selected leg for the No ACL group. Direct contact–related new ACL injuries were excluded from this analysis (n = 8). We performed 2-way analyses of variance, with sport (2 levels: handball and soccer) and injury status as independent variables and knee-specific biomechanical variables as the dependent variables. In the first analysis, the independent variable “injury status” comprised 2 levels (players with and without a previous ACL injury), and in the second analysis, it comprised 4 levels (all 4 groups presented in Figure 1). For the second analysis, significant effects were followed by post hoc tests. In the current study, main effects are the effects of injury status on knee biomechanics for soccer and handball players combined, whereas simple main effects are the effects of injury status on knee biomechanics for either soccer or handball players alone. Interaction effects occurred when the effect of injury status on knee biomechanics was different for soccer and handball players. To detect statistically significant differences between injury groups, non–statistically significant interactions were followed by main effects and significant interactions by simple main effects. If an interaction effect was significant, simple main effects but not main effects are reported.^11,29^ Main effects are presented in the tables, and simple main effects are reported in the text. The level of significance was set a priori at *P*≤ .05. Results are presented as mean ± SD. For main effects and simple main effects, the *P* value and mean difference (MD) with 95% CI are reported.

For the prospective cohort part of this study, we investigated whether the knee-specific variables were associated with future, secondary ACL injury. Only players with a history of an ACL injury (ie, Prev/New ACL group and Prev ACL group) were included in this analysis, and direct contact–related new ACL injuries were excluded (n = 4). We generated separate binomial logistic regression models, one for each of the proposed knee-specific risk factors, with new, secondary ACL injury as the outcome measure. This approach was chosen rather than multivariable logistic regression, because ≥10 new injuries are recommended for each variable included in the model.^49^ We performed separate analyses for the ipsilateral knee and ipsilateral reinjury as well as for the contralateral knee and contralateral injury. In the primary analysis, we adjusted for the effect of sport (handball and soccer), and in the secondary analysis we generated a model adjusting for the effect of the time that had passed from the injury to the testing day (in years). Data are presented as odds ratio (OR) per 1-unit change (corresponding to the change in the odds of sustaining a secondary ACL injury for each increase in 1 unit of the independent variable) with 95% CI.

## Results

During the follow-up period, 15 players sustained a secondary noncontact ACL injury (7 ipsilateral and 8 contralateral), including the players with missing data (n = 1), and 51 players, including the players with missing data (n = 5), sustained a primary noncontact ACL injury (Figure 1). The ACL injuries occurred a mean 1.8 ± 1.7 years after baseline testing with a range of 0.03 to 7.2 years (Prev/New ACL group, 1.4 ± 1.3 years; New ACL group, 2.0 ± 1.8 years). The mean cutting angles were 68°± 26° and 66°± 14° for soccer and handball players, respectively.

### Differences Between Players With and Without a Previous ACL Injury

Players with a previous ACL injury were significantly older and heavier than injury-free players, but no differences were found for height (Table 1). However, handball players with a previous ACL injury were significantly taller than injury-free handball players (MD, 2.0 cm; 95% CI, 0.1-3.9 cm).

**Table 1:** Comparison of Participant Characteristics Between Players With and Without a Previous ACL Injury (n = 746)^*a*^

	Previous ACL Injury (n = 67)	No Previous ACL Injury (n = 679)	*P*	MD (95% CI)
Age, y	23.2 ± 4.1	20.6 ± 3.9	<.001	2.6^*b*^ (1.6 to 3.6)
Body mass, kg	68.0 ± 7.8	66.1 ± 7.7	.041	1.9^*b*^ (0.1 to 3.7)
Height, cm	170.5 ± 6.4	169.5 ± 6.3	.158	1.1 (–0.4 to 2.6)

a Values are expressed as mean ± SD unless otherwise noted. ACL, anterior cruciate ligament; MD, mean difference.

b Significant mean difference (*P*≤ .05).

Comparing the ipsilateral leg of players who had a previous ACL injury versus the legs of injury-free players, we found significant main effects for 5 of the 7 primary knee-specific variables, revealing lower knee abduction angles and knee joint moments in addition to lower approach speeds in players with a previous ACL injury (Table 2).

**Table 2:** Comparison of Knee Biomechanics and Cutting Technique Variables Between Players With and Without a Previous ACL Injury (n = 744 for angles and approach speed; n = 702 for joint moments)^*a*^

Variable	Previous ACL Injury, Ipsilateral Leg (n = 64)	No Previous ACL Injury, Random Leg (n = 679)	*P*	MD (95% CI)	Previous ACL Injury, Contralateral Leg (n = 65)	No Previous ACL Injury, Random Leg (n = 679)	*P*	MD (95% CI)
Knee flexion angle at IC, deg	25.1 ± 8.3	24.3 ± 7.9	.310	0.9 (–0.8 to 2.5)	25.3 ± 8.9	24.0 ± 7.6	.121	1.3 (–0.3 to 2.9)
Knee flexion angle peak, deg	62.4 ± 7.1	62.7 ± 6.7	.743	0.3 (–1.5 to 2.0)	63.7 ± 6.5	62.9 ± 6.7	.335	0.8 (–0.9 to 2.6)
Knee abduction angle at IC, deg	3.4 ± 4.4	4.8 ± 4.3	.016	1.4^*b*^ (0.3 to 2.5)	4.6 ± 4.2	4.8 ± 4.3	.634	0.3 (–0.8 to 1.4)
Knee abduction angle peak, deg	9.3 ± 5.2	10.8 ± 5.2	.028	1.5^*b*^ (0.2 to 2.9)	10.2 ± 4.9	11.0 ± 5.1	.207	0.8 (–0.5 to 2.2)
Knee flexion moment peak, N·m/kg^-1^	2.56 ± 0.62	2.89 ± 0.58	<.001	0.33^*b*^ (0.18 to 0.48)	2.87 ± 0.71	2.89 ± 0.59	.818	0.02 (–0.14 to 0.18)
Knee abduction moment peak, N·m/kg^-1^	1.40 ± 0.50	1.67 ± 0.55	<.001	0.27^*b*^ (0.12 to 0.41)	1.59 ± 0.52	1.67 ± 0.54	.266	0.08 (–0.06 to 0.23)
Knee internal rotation moment peak, N·m/kg^-1^	0.18 ± 0.19	0.24 ± 0.21	.031	0.06^*b*^ (0.01 to 0.12)	0.29 ± 0.26	0.23 ± 0.20	.056	0.06 (–0.00 to 0.11)
Approach speed at IC, m·s^-1^	2.96 ± 0.43	3.11 ± 0.50	.004	0.16^*b*^ (0.05 to 0.26)	3.06 ± 0.47	3.11 ± 0.50	.327	0.05 (–0.05 to 0.16)

a Values are expressed as mean ± SD unless otherwise noted. ACL, anterior cruciate ligament; IC, initial contact; MD, mean difference.

b Significant mean difference (*P*≤ .05).

Comparing the contralateral leg of players who had a previous ACL injury versus legs of injury-free players, we found no significant main effects for any of the variables analyzed (Table 2). However, soccer players with a previous ACL injury showed significantly larger knee flexion angles at IC compared with injury-free soccer players (MD, 2.7°; 95% CI, 0.2°-5.2°), and handball players with a previous ACL injury showed significantly larger peak knee internal rotation moments than injury-free handball players (MD, 0.09 N·m/kg; 95% CI, 0.01-0.16 N·m/kg).

Main effects for side-to-side differences showed that players with a previous ACL injury exhibited larger asymmetry in knee flexion angle at IC (MD, 1.1°; 95% CI, 0.2°-2.1°), peak knee flexion angle (MD, 1.4°; 95% CI, 0.5°-2.3°), and cutting angle (MD, 3.4°; 95% CI, 1.2°-5.6°) than injury-free players. Soccer players with a previous ACL injury had larger asymmetry in knee abduction angle at IC compared with injury-free soccer players (MD, 1.3°; 95% CI, 0.6°-2.5°).

### Differences Between Players Who Went On to Sustain a Secondary ACL Injury and Those Who Did Not

Comparing players with a previous ACL injury who went on to sustain an ipsilateral reinjury during follow-up (Prev/New ACL group) with the remaining 3 groups, we found the Prev/New ACL group to be heavier and taller than all other groups (Appendix Table A1, available in the online version of this article). Moreover, the Prev/New ACL group had significantly larger knee flexion angles at IC as well as lower peak knee abduction moments than the New ACL group (Appendix Table A1, available online). Side-to-side differences were significant for knee flexion angle at IC, showing larger asymmetry in the Prev/New ACL group than the No ACL group (MD, 3.7°; 95% CI, 0.2°-7.1°).

Comparing players with a previous ACL injury who went on to sustain a new contralateral injury during follow-up (Prev/New ACL group) with the remaining 3 groups, we observed that the Prev/New ACL group was older than the New ACL group and the No ACL group (Appendix Table A2, available online). Also, the Prev/New ACL group had lower approach speeds at IC than the New ACL group (Appendix Table A2, available online). Side-to-side differences were significant for knee flexion angle at IC, showing larger asymmetry in the Prev/New ACL group than the Prev ACL group and the No ACL group (MD, 3.1°-3.2°; 95% CI, 0.1°-6.1°).

### Association Between Knee Biomechanics and Future Secondary ACL Injury

The 7 knee biomechanical variables (Table 3) as well as the side-to-side differences in these variables were not significantly associated with future secondary ipsilateral ACL injury. Only body mass, height, and the adjustment factors “time since injury” and “sport” had significant odds ratios in some of the models, indicating an increased risk with increasing body mass and height, an increased risk when playing soccer compared with handball, and a decreased risk with increasing time since injury (Table 3).

**Table 3:** Adjusted Odds Ratios (per 1-unit change) for Participant Characteristics, Knee Biomechanics, and Cutting Technique Variable^*a*^

	Secondary Ipsilateral ACL Injury (n = 55; 6 with a new secondary ipsilateral ACL injury and 49 without)				Secondary Contralateral ACL Injury (n = 58; 8 with a new secondary contralateral ACL injury and 50 without)			
	Adjusted for Sport		Adjusted for Time Since Injury		Adjusted for Sport		Adjusted for Time Since Injury	
	Risk Factor	Adjustment Factor	Risk Factor	Adjustment Factor	Risk Factor	Adjustment Factor	Risk Factor	Adjustment Factor
Age, y	0.84 (0.64-1.10)	3.43 (0.55-21.23)	0.81 (0.57-1.15)	0.24 (0.06-1.01)	1.07 (0.90-1.28)	0.99 (0.21-4.63)	1.11 (0.91-1.35)	0.83 (0.57-1.19)
Body mass, kg	1.16^*b*^ (1.02-1.33)	9.39^*b*^ (1.03-85.78)	1.09 (0.96-1.23)	0.32 (0.10-1.00)	1.10 (0.97-1.24)	2.06 (0.32-13.32)	1.07 (0.96-1.19)	0.89 (0.62-1.28)
Height, cm	1.36^*b*^ (1.02-1.82)	28.99^*b*^ (1.41-598.21)	1.13 (0.96-1.33)	0.28^*b*^ (0.08-0.99)	1.03 (0.89-1.20)	1.19 (0.19-7.62)	1.03 (0.91-1.17)	0.87 (0.61-1.24)
Knee flexion angle at IC, deg	1.14 (0.95-1.37)	0.81 (0.07-10.07)	1.11 (0.96-1.28)	0.26 (0.07-1.04)	0.94 (0.83-1.07)	1.87 (0.22-15.58)	0.96 (0.87-1.05)	0.86 (0.59-1.25)
Knee flexion angle peak, deg	1.09 (0.96-1.23)	2.17 (0.32-14.66)	1.11 (0.98-1.27)	0.22^*b*^ (0.05-1.00)	0.94 (0.83-1.06)	0.98 (0.21-4.66)	0.93 (0.82-1.05)	0.87 (0.62-1.24)
Knee abduction angle at IC, deg	0.97 (0.79-1.18)	3.35 (0.54-20.71)	0.97 (0.76-1.22)	0.28^*b*^ (0.08-0.98)	1.14 (0.93-1.39)	0.76 (0.16-3.68)	1.15 (0.94-1.41)	0.86 (0.61-1.23)
Knee abduction angle peak, deg	1.00 (0.85-1.18)	3.15 (0.52-19.27)	0.98 (0.79-1.22)	0.29^*b*^ (0.08-0.98)	1.08 (0.92-1.26)	1.09 (0.22-5.41)	1.09 (0.94-1.26)	0.86 (0.61-1.22)
Knee flexion moment peak, N·m/kg^-1^	0.42 (0.07-2.41)	4.98 (0.51-49.14)	0.42 (0.07-2.60)	0.23 (0.05-1.14)	0.94 (0.33-2.68)	0.81 (0.17-3.85)	1.00 (0.33-3.00)	0.89 (0.63-1.26)
Knee abduction moment peak, N·m/kg^-1^	0.11 (0.01-2.85)	10.26 (0.73-144.28)	0.20 (0.01-4.36)	0.20 (0.04-1.13)	2.29 (0.57-9.23)	0.79 (0.17-3.79)	3.57 (0.68-18.90)	0.84 (0.59-1.19)
Knee internal rotation moment peak, N·m/kg^-1^	13.69 (0.43-434.12)	5.27 (0.52-53.04)	17.64 (0.22-1442.42)	0.20 (0.03-1.18)	0.70 (0.03-18.67)	0.79 (0.16-3.86)	1.15 (0.04-36.46)	0.89 (0.63-1.26)
Cutting angle, deg	1.06 (0.98-1.14)	0.89 (0.06-13.15)	1.04 (0.99-1.09)	0.32 (0.10-1.08)	1.00 (0.96-1.05)	0.90 (0.17-4.68)	1.00 (0.97-1.04)	0.88 (0.62-1.25)
Approach speed at IC, m·s^-1^	0.64 (0.03-14.02)	2.54 (0.20-33.14)	0.55 (0.05-6.61)	0.29^*b*^ (0.09-0.99)	0.30 (0.03-2.94)	0.45 (0.05-3.73)	0.68 (0.12-3.92)	0.90 (0.63-1.29)

a Values are expressed as odds ratio (95% CI). The analyses adjusted either for the effect of sport (handball or soccer) or for the effect of the time that had passed from the injury to the testing day (in years). ACL, anterior cruciate ligament; IC, initial contact.

b Odds ratio significantly (*P*≤ .05) different from 1.

None of the 7 potential knee biomechanical risk factors was significantly associated with future secondary contralateral ACL injury (Table 3). However, side-to-side difference in knee flexion angle at IC had a significant odds ratio in both models (OR, 1.20-1.21; 95% CI, 1.02-1.44), indicating an increased risk with increasing asymmetry.

## Discussion

This study aimed to assess potential knee-specific biomechanical risk factors for secondary ACL injury by means of combining a prospective cohort design with a cross-sectional design. When players with a previous ACL injury performed a cutting maneuver with their ipsilateral leg, they exhibited lower knee abduction angles, lower peak knee joint moments around all axes of rotation, and lower approach speeds, compared with injury-free players. When players performed the cut with their contralateral leg, no differences were evident. Side-to-side differences in knee flexion angle were larger in players with a previous ACL injury. Knee biomechanical variables in players who went on to sustain a secondary ACL injury did not differ substantially from players with a previous ACL injury, players who went on to sustain a primary ACL injury, and injury-free players. Notably, players who went on to sustain an ipsilateral reinjury displayed larger knee flexion angles at IC and lower peak knee abduction moments than players who went on to sustain a primary ACL injury. Finally, not knee biomechanical parameters, but body mass, height, and the time that had passed since the injury were significantly associated with future ipsilateral reinjury. The only variable associated with secondary contralateral ACL injury was side-to-side difference in knee flexion angle at IC.

### Do Players With a Previous ACL Injury Cut Differently?

When players with an ACL injury history performed the cutting maneuver with their ipsilateral leg, 5 of the 7 knee-specific biomechanical variables, as well as approach speed, differed compared with injury-free players. Players with a previous ACL injury exhibited slightly slower approach speeds (5% difference compared with injury-free players), implying that they still seemed to restrain themselves when performing a cut with their ipsilateral leg. In contrast, Lee et al^30^ did not find differences in approach speed during a planned 45° cutting task between injured and uninjured players approximately 2 years after injury. However, the approach speeds in their study were slower than those in our study (2.7-2.8 vs 3.0-3.1 m·s^-1^, respectively), despite wider cutting angles (45° vs ~67°), implying submaximal effort during cutting, which might have masked between-group differences. In agreement with our finding, previous studies on male athletes 9 months after ACL reconstruction revealed slower approach speeds when the athletes performed a planned and unplanned 90° cut with their ipsilateral leg compared with their contralateral leg.^7,24^

Surprisingly, ipsilateral knee abduction angles at IC and peak knee abduction angles were slightly lower in players with an ACL injury history (1.4° and 1.5° difference compared with injury-free players, respectively). Lower knee abduction angles are commonly considered to be part of a less risky movement pattern.^1^ It can be speculated that these favorable knee kinematics might be related to rehabilitation training after the previous ACL injury or simply attributable to unintentional movement modifications to reduce ACL load. In contrast to our results, Stearns and Pollard^51^ found larger mean knee abduction angles in female soccer players performing a planned 45° cutting task 4 years after ACL reconstruction compared with uninjured players, whereas Lee et al^30^ found no differences between groups during a planned 45° cutting task 2 years after ACL reconstruction.

Further, we found 19% lower ipsilateral peak knee abduction moments, 13% lower peak knee flexion moments, and 33% lower peak knee internal rotation moments in players with a previous ACL injury compared with uninjured players. This indicates that there is likely less loading on the ACL after injury. The reduced knee joint moments might be explained by the lower approach speed causing lower ground-reaction forces during the cutting maneuver or by a change in the moment distribution between the knee, hip, and ankle joints (ie, decreased knee loading by compensatory increased loading on the adjacent joints).^40^ Secondary analyses of our data indicated that a combination of both a reduced peak vertical ground-reaction force and increased peak hip flexion moment (probably caused by increased trunk forward lean) might be explanatory, even though these differences were not significant. The reduced peak knee abduction moments can likely also be explained by the reduced peak knee abduction angles. In contrast to our results, Stearns and Pollard^51^ found larger peak knee abduction moments during planned 45° cutting in previously injured players compared with uninjured players. In agreement with our results, Bush-Joseph et al^4^ found lower peak knee flexion moments during a planned jog and cut task in participants with ACL reconstruction 22 months after surgery compared with healthy controls. Studies assessing between-limb differences during planned and unplanned 90° cutting after ACL reconstruction found reduced knee joint moments in all 3 planes of motion as well as reduced resultant ground-reaction forces in the ipsilateral limb compared with the contralateral limb 9 months after surgery.^7,24,34^ In agreement with our findings from a sport-specific cutting task, recent systematic reviews and meta-analyses reported reduced ipsilateral knee flexion moments during single-leg landing,^20^ double-leg landing,^31^ running,^41^ walking,^21^ and stair negotiation^21^ as well as reduced vertical ground-reaction forces during single- and double-leg landing tasks^31^ in patients with ACL reconstruction compared with healthy controls, with differences still being present years after surgery. Differences in transverse and frontal plane knee joint kinetics were less marked and more conflicting. The unloading of the ipsilateral knee joint in the sagittal plane after ACL reconstruction might be related to physical factors, such as reduced ipsilateral quadriceps strength^41,50^ and quadriceps activation, which can persist for years after surgery,^32^ or psychological factors, such as fear avoidance or kinesiophobia.^8^ Quadriceps avoidance movement patterns have also been postulated to be a compensatory strategy to decrease anterior tibial translation and thus ACL loading.^16^

The results of this study imply that players with a previous ACL injury unload their ipsilateral knee, unintentionally or intentionally, even though the injury happened a mean 3.6 years previously. On the basis of this finding, one might assume that these players are less likely to sustain a new ACL injury in their ipsilateral knee, but in fact we know that they have an increased risk.^53^ This finding intuitively questions the importance of movement biomechanics for ipsilateral reinjury risk. However, one might speculate that knee unloading reflects reduced muscle strength, reduced neuromuscular control, or increased knee laxity after ACL reconstruction, which potentially can cause unfavorable movement kinematics (eg, valgus collapse) with high knee forces in uncontrolled, unanticipated, and high-intensity match situations, thus increasing susceptibility to injury. In this sense, theoretically, unloading of the knee might be a risk factor for ipsilateral reinjury. Potentially, increasing match-specificity even more during laboratory cutting test scenarios might reveal such unfavorable movement patterns. However, previous research does not support this notion.^33^

When players with an ACL injury history performed the cutting maneuver with their contralateral leg, no differences were found compared with legs of injury-free players. This implies that players with an ACL injury history load their contralateral knee to a similar extent to injury-free players. This finding might lead one to assume that these players are similarly likely to sustain a new ACL injury in their contralateral leg compared with injury-free players, but we know that they have an increased risk.^53^ This finding brings into question the importance of movement biomechanics for contralateral ACL injury risk. To our knowledge, no studies have compared biomechanics of the contralateral knee of ACL-injured players with those of healthy controls during cutting maneuvers or other unilateral tasks, even though contralateral ACL injury rates are as least as high as ipsilateral reinjury rates.^53^ During bilateral tasks, such as vertical drop jumps, athletes with ACL injury have displayed increased compensatory loading on the contralateral leg compared with healthy controls.^43,50^ This might be a contributing factor to the increased risk of contralateral ACL injury. However, we could not replicate these findings during cutting maneuvers in our data. We did note a tendency toward increased peak knee internal rotation moments in players with a previous ACL injury compared with injury-free players (*P* = .056; 26% difference), a difference that reached significance for handball players (*P* = .020; 35% difference).

In agreement with previous research on male athletes during unplanned 90° cutting,^25^ we found 1.1° to 1.4° larger between-limb asymmetry in knee flexion angle in previously injured players compared with healthy players. This asymmetry in the players with ACL injury was directed toward larger knee flexion angles in the contralateral leg than in the ipsilateral leg (Table 2). Greater biomechanical between-limb asymmetry in athletes with ACL injury versus uninjured athletes has commonly been reported in the literature,^19,25^ but whether and how these alterations relate to future secondary ACL injury remains unclear.

### Do Players Who Go On to Sustain a Secondary ACL Injury Cut Differently?

None of the 7 knee-specific biomechanical variables differed between players who went on to sustain a secondary ACL injury and those who remained with a primary ACL injury only. However, players who went on to sustain a secondary contralateral ACL injury displayed 3.1° larger asymmetry in knee flexion angle at IC compared with players who had a primary ACL injury only, most likely caused by larger knee flexion angles at IC in the ipsilateral leg than the contralateral leg (Appendix Tables A1 and A2, available online). To our knowledge, only 2 previous studies have compared cutting biomechanics between players with and without a new secondary ACL injury. These 2 studies are from the same large cohort of 1045 male, multidirectional field sport athletes with unilateral ACL reconstruction and were purposefully divided into ipsilateral reinjuries and contralateral injuries.^22,23^ In correspondence with our findings, the investigators found no differences in ipsilateral knee biomechanics and knee asymmetry during unplanned and planned 90° cutting tasks between the 31 players who experienced an ipsilateral reinjury during follow-up and 57 matched controls without secondary ACL injury.^23^ Comparing contralateral leg biomechanics and leg asymmetry during unplanned and planned 90° cutting tasks between the 55 players who experienced a contralateral ACL injury during follow-up and 60 matched controls without secondary ACL injury, the investigators found no differences in knee biomechanics.^22^ Collectively, it appears that knee biomechanical characteristics during cutting tasks do not differ distinctly between previously injured players who sustain a secondary ACL injury and those who do not.

To the best of our knowledge, this is the first study to compare knee biomechanics between players who went on to sustain a secondary ACL injury, players who went on to sustain a primary ACL injury, and injury-free players. Interestingly, players who sustained an ipsilateral reinjury during follow-up displayed 5.8° larger knee flexion angles at IC and 68% lower peak knee abduction moments than players who sustained a primary ACL injury during follow-up, indicating that risk factors for secondary ipsilateral and primary ACL injury might differ. Players who sustained a secondary contralateral ACL injury did not show differences in the 7 primary knee biomechanical variables compared with the other groups yet had 12% slower approach speeds than players who went on to sustain a primary ACL injury. This aligns with our previous findings and might indicate that these players restrain themselves when performing a cutting maneuver. Finally, players with an ipsilateral reinjury and players with a new contralateral injury displayed larger asymmetry in knee flexion angle at IC than injury-free players (differences of 3.7° and 3.2°, respectively), most likely caused by larger knee flexion angles at IC in the ipsilateral leg than the contralateral leg (Appendix Tables A1 and A2, available online).

### Are Knee Biomechanical Parameters Associated With Future Secondary ACL Injury?

The 7 primary knee-specific biomechanical variables were not significantly associated with future secondary ACL injury in our study. This coincides with previous research on male athletes assessing the ability of biomechanical variables during unplanned and planned 90° cutting maneuvers to predict future ACL reinjury^22^ and future contralateral ACL injury.^23^ Further, King et al^22^ reported limited ability (area under the curve, 0.75) of 3 biomechanical variables to prospectively predict ACL reinjury during unplanned 90° cutting; the 3 variables were symmetry of center of mass position, pelvic drop, and trunk side flexion, which were chosen per the largest effect sizes of the identified differences between groups. Unlike King et al,^23^ we observed a significant association between side-to-side difference in knee flexion angle at IC and the risk of future secondary contralateral ACL injury, suggesting a 20% to 21% higher risk for each degree of increased asymmetry. It would be interesting to compare our results with the results of prospective studies assessing associations between cutting biomechanics and future primary ACL injury. However, to the best of our knowledge, there are no existing studies in this specific context. Collectively, based on the currently available research, knee biomechanical parameters during cutting maneuvers do not seem to be distinctly associated with an increased risk of future secondary ACL injury in a controlled laboratory environment.

Interestingly, in the sport-adjusted model, body mass and height were significantly associated with future secondary ipsilateral ACL injury, indicating a 16% and 36% higher risk per 1-kg and 1-cm increase, respectively. This aligns with our previous findings showing that players with an ACL injury history were 3% heavier than injury-free players, and handball players with a previous ACL injury were 1% taller than injury-free handball players. We also performed secondary analyses of body mass index, finding no differences among any of the groups and no associations with future secondary ipsilateral ACL injury. This is consistent with the findings from Cronström et al^5^ demonstrating no association between body mass index and graft rupture. Hence, height-driven increases in body mass seem to be a risk factor for secondary ipsilateral ACL injury. Further, the adjustment factor “time since injury” was significantly associated with secondary ipsilateral ACL injury in 5 of our models. Per year that had passed since the injury, the risk to sustain an ACL reinjury decreased by 71% to 78%, indicating that time seems to be a protective factor. This coincides with the results of a review article by Nagelli and Hewett^38^ ascertaining that secondary ACL injury risk is greatest within the first 2 years after ACL reconstruction, gradually abating over this time period.

### Limitations

First, this study had a limited sample size with 14 players in the Prev/New ACL group, including 6 ACL reinjuries and 8 contralateral injuries, restricting the statistical power of the analyses involving this group. This reduced the chance of detecting true differences between the groups and true associations between knee biomechanics and future secondary ACL injury. Second, our participants with a previous ACL injury were heterogeneous with respect to the time that had passed since the injury. Although we accounted for this statistically in the binominal logistic regression, future research should investigate whether and how cutting biomechanical parameters change with time after ACL injury. Third, the significant association between the time since injury and secondary ipsilateral ACL injury might have been affected by a survivor bias. Potentially, players who sustained their ACL injury a long time ago were older and thus more likely to end their sporting career sooner than those who became injured within recent years, thereby reducing the occurrence of secondary ACL injuries in the former group. Unfortunately, we do not have data on points in time at which players ended their sporting career. Fourth, our analyses were limited to discrete data points. Although this may have led to the loss of potentially valuable information compared with functional data analysis, it has the benefit of reducing the risk of type I error. Fifth, during the data collection we upgraded our camera system as described in the Methods section. Although the newer system is expected to provide slightly higher measurement accuracy, it is unlikely to have affected the outcome of this study. Sixth, although the data collection and biomechanical calculations were completed several years ago, it is important to acknowledge that during this period technological advancements have introduced new computational biomechanics approaches that were not considered in the current data set.

## Conclusion

Knee biomechanics during side-step cutting maneuvers could not be identified as a risk factor for secondary ACL injury in female elite team-ball sport athletes. First, players with a previous ACL injury showed favorable knee kinematics and reduced knee loading when performing the cutting maneuver with their ipsilateral leg, and no differences were evident when they cut with their contralateral leg compared with injury-free players. Hence, players with a previous ACL injury did not show a more unfavorable movement or loading pattern of either knee. Second, none of the 7 primary knee-specific biomechanical variables was associated with future secondary ACL injury.

## Supplemental Material

sj-pdf-1-ajs-10.1177_03635465241234255 – Supplemental material for Knee Biomechanics During Cutting Maneuvers and Secondary ACL Injury Risk: A Prospective Cohort Study of Knee Biomechanics in 756 Female Elite Handball and Soccer Players Supplemental material, sj-pdf-1-ajs-10.1177_03635465241234255 for Knee Biomechanics During Cutting Maneuvers and Secondary ACL Injury Risk: A Prospective Cohort Study of Knee Biomechanics in 756 Female Elite Handball and Soccer Players by Lasse Mausehund and Tron Krosshaug in The American Journal of Sports Medicine

## References

[1] Alentorn-Geli E Myer GD Silvers HJ , et al. Prevention of non-contact anterior cruciate ligament injuries in soccer players, part 1: mechanisms of injury and underlying risk factors. Knee Surg Sports Traumatol Arthrosc. 2009;17(7):705-729. 19452139 10.1007/s00167-009-0813-1

[2] Bahr R Krosshaug T . Understanding injury mechanisms: a key component of preventing injuries in sport. Br J Sports Med. 2005;39(6):324. 15911600 10.1136/bjsm.2005.018341 PMC1725226

[3] Bell AL Pedersen DR Brand RA . A comparison of the accuracy of several hip center location prediction methods. J Biomech. 1990;23(6):617-621. 2341423 10.1016/0021-9290(90)90054-7

[4] Bush-Joseph CA Hurwitz DE Patel RR , et al. Dynamic function after anterior cruciate ligament reconstruction with autologous patellar tendon. Am J Sports Med. 2001;29(1):36-41. 11206254 10.1177/03635465010290011101

[5] Cronström A Tengman E Häger CK . Return to sports: a risky business? A systematic review with meta-analysis of risk factors for graft rupture following ACL reconstruction. Sports Med. 2023;53(1):91-110. 36001289 10.1007/s40279-022-01747-3 PMC9807539

[6] Cronström A Tengman E Häger CK . Risk factors for contra-lateral secondary anterior cruciate ligament injury: a systematic review with meta-analysis. Sports Med. 2021;51(7):1419-1438. 33515391 10.1007/s40279-020-01424-3 PMC8222029

[7] Daniels KAJ Drake E King E Strike S . Whole-body change-of-direction task execution asymmetries after anterior cruciate ligament reconstruction. J Appl Biomech. 2021;37(3):176-181. 33482630 10.1123/jab.2020-0110

[8] Dashti Rostami K Nabavinik M Naderi E . Relationship between kinesiophobia and vertical ground reaction force in anterior cruciate ligament reconstructed and deficient patients during landing task. J Rehab Sci Res. 2021;8(1):25-30.

[9] Fältström A Hägglund M Kvist J . Patient-reported knee function, quality of life, and activity level after bilateral anterior cruciate ligament injuries. Am J Sports Med. 2013;41(12):2805-2813. 24007758 10.1177/0363546513502309

[10] Faunø P Wulff Jakobsen B . Mechanism of anterior cruciate ligament injuries in soccer. Int J Sports Med. 2006;27(1):75-79. 16388446 10.1055/s-2005-837485

[11] Field A . Discovering Statistics Using SPSS (and Sex and Drugs and Rock ’n’ Roll). 3rd ed. Sage; 2009.

[12] Filbay SR Ackerman IN Russell TG Macri EM Crossley KM . Health-related quality of life after anterior cruciate ligament reconstruction: a systematic review. Am J Sports Med. 2014;42(5):1247-1255. 24318609 10.1177/0363546513512774

[13] Francesco Della V Matthew B Alberto G , et al. Systematic video analysis of ACL injuries in professional male football (soccer): injury mechanisms, situational patterns and biomechanics study on 134 consecutive cases. Br J Sports Med. 2020;54(23):1423. 32561515 10.1136/bjsports-2019-101247

[14] Grassi A Ardern CL Marcheggiani Muccioli GM Neri MP Marcacci M Zaffagnini S . Does revision ACL reconstruction measure up to primary surgery? A meta-analysis comparing patient-reported and clinician-reported outcomes, and radiographic results. Br J Sports Med. 2016;50(12):716-724. 26809259 10.1136/bjsports-2015-094948

[15] Grassi A Zaffagnini S Marcheggiani Muccioli GM Neri MP Della Villa S Marcacci M . After revision anterior cruciate ligament reconstruction, who returns to sport? A systematic review and meta-analysis. Br J Sports Med. 2015;49(20):1295-1304. 26062956 10.1136/bjsports-2014-094089

[16] Hajizadeh M Hashemi Oskouei A Ghalichi F Sole G . Knee kinematics and joint moments during stair negotiation in participants with anterior cruciate ligament deficiency and reconstruction: a systematic review and meta-analysis. PM R. 2016;8(6):563-579.e1. 10.1016/j.pmrj.2016.01.014 26872590

[17] Hart HF Culvenor AG Collins NJ , et al. Knee kinematics and joint moments during gait following anterior cruciate ligament reconstruction: a systematic review and meta-analysis. Br J Sports Med. 2016;50(10):597-612. 26265562 10.1136/bjsports-2015-094797

[18] Hewett TE Di Stasi SL Myer GD . Current concepts for injury prevention in athletes after anterior cruciate ligament reconstruction. Am J Sports Med. 2013;41(1):216-224. 23041233 10.1177/0363546512459638 PMC3592333

[19] Ithurburn MP Paterno MV Ford KR Hewett TE Schmitt LC . Young athletes with quadriceps femoris strength asymmetry at return to sport after anterior cruciate ligament reconstruction demonstrate asymmetric single-leg drop-landing mechanics. Am J Sports Med. 2015;43(11):2727-2737. 26359376 10.1177/0363546515602016

[20] Johnston PT McClelland JA Webster KE . Lower limb biomechanics during single-leg landings following anterior cruciate ligament reconstruction: a systematic review and meta-analysis. Sports Med. 2018;48(9):2103-2126. 29949109 10.1007/s40279-018-0942-0

[21] Kaur M Ribeiro DC Theis JC Webster KE Sole G . Movement patterns of the knee during gait following ACL reconstruction: a systematic review and meta-analysis. Sports Med. 2016;46(12):1869-1895. 26936269 10.1007/s40279-016-0510-4

[22] King E Richter C Daniels KAJ , et al. Biomechanical but not strength or performance measures differentiate male athletes who experience ACL reinjury on return to level 1 sports. Am J Sports Med. 2021;49(4):918-927. 33617291 10.1177/0363546520988018 PMC9677345

[23] King E Richter C Daniels KAJ , et al. Can biomechanical testing after anterior cruciate ligament reconstruction identify athletes at risk for subsequent ACL injury to the contralateral uninjured limb? Am J Sports Med. 2021;49(3):609-619. 33560866 10.1177/0363546520985283 PMC9938948

[24] King E Richter C Franklyn-Miller A , et al. Biomechanical but not timed performance asymmetries persist between limbs 9 months after ACL reconstruction during planned and unplanned change of direction. J Biomech. 2018;81:93-103. 30322642 10.1016/j.jbiomech.2018.09.021

[25] King E Richter C Franklyn-Miller A Wadey R Moran R Strike S . Back to normal symmetry? Biomechanical variables remain more asymmetrical than normal during jump and change-of-direction testing 9 months after anterior cruciate ligament reconstruction. Am J Sports Med. 2019;47(5):1175-1185. 30943079 10.1177/0363546519830656

[26] Kristianslund E Faul O Bahr R Myklebust G Krosshaug T . Sidestep cutting technique and knee abduction loading: implications for ACL prevention exercises. Br J Sports Med. 2014;48(9):779-783. 23258848 10.1136/bjsports-2012-091370

[27] Kristianslund E Krosshaug T van den Bogert AJ . Effect of low pass filtering on joint moments from inverse dynamics: implications for injury prevention. J Biomech. 2012;45(4):666-671. 22227316 10.1016/j.jbiomech.2011.12.011

[28] Krosshaug T Steffen K Kristianslund E , et al. The vertical drop jump is a poor screening test for ACL injuries in female elite soccer and handball players: a prospective cohort study of 710 athletes. Am J Sports Med. 2016;44(4):874-883. 26867936 10.1177/0363546515625048

[29] Laerd-Statistics. Two-way ANOVA using SPSS Statistics: statistical tutorials and software guides. Accessed October 3, 2023. https://statistics.laerd.com/

[30] Lee SP Chow JW Tillman MD . Persons with reconstructed ACL exhibit altered knee mechanics during high-speed maneuvers. Int J Sports Med. 2014;35(6):528-533. 24408765 10.1055/s-0033-1358466

[31] Lepley AS Kuenze CM . Hip and knee kinematics and kinetics during landing tasks after anterior cruciate ligament reconstruction: a systematic review and meta-analysis. J Athl Train. 2018;53(2):144-159. 29350551 10.4085/1062-6050-334-16 PMC5842905

[32] Lisee C Lepley AS Birchmeier T O’Hagan K Kuenze C . Quadriceps strength and volitional activation after anterior cruciate ligament reconstruction: a systematic review and meta-analysis. Sports Health. 2019;11(2):163-179. 30638441 10.1177/1941738118822739 PMC6391557

[33] Mai P Bill K Glöckler K , et al. Unanticipated fake-and-cut maneuvers do not increase knee abduction moments in sport-specific tasks: implication for ACL injury prevention and risk screening. Front Sports Act Living. 2022;4:983888. 36439622 10.3389/fspor.2022.983888 PMC9685612

[34] Miles JJ McGuigan PM King E Daniels KAJ . Biomechanical asymmetries differ between autograft types during unplanned change of direction after ACL reconstruction. Scand J Med Sci Sports. 2022;32(8):1236-1248. 35419809 10.1111/sms.14166

[35] Mok KM Bahr R Krosshaug T . Reliability of lower limb biomechanics in two sport-specific sidestep cutting tasks. Sports Biomech. 2018;17(2):157-167. 28281390 10.1080/14763141.2016.1260766

[36] Myklebust G Maehlum S Engebretsen L Strand T Solheim E . Registration of cruciate ligament injuries in Norwegian top level team handball: a prospective study covering two seasons. Scand J Med Sci Sports. 1997;7(5):289-292. 9338947 10.1111/j.1600-0838.1997.tb00155.x

[37] Myklebust G Maehlum S Holm I Bahr R . A prospective cohort study of anterior cruciate ligament injuries in elite Norwegian team handball. Scand J Med Sci Sports. 1998;8(3):149-153. 9659675 10.1111/j.1600-0838.1998.tb00185.x

[38] Nagelli CV Hewett TE . Should return to sport be delayed until 2 years after anterior cruciate ligament reconstruction? Biological and functional considerations. Sports Med. 2017;47(2):221-232. 27402457 10.1007/s40279-016-0584-z PMC5226931

[39] Nilstad A Petushek E Mok KM Bahr R Krosshaug T . Kiss goodbye to the “kissing knees”: no association between frontal plane inward knee motion and risk of future non-contact ACL injury in elite female athletes. Sports Biomech. 2023;22(1):65-79. 33906580 10.1080/14763141.2021.1903541

[40] Oberländer KD Brüggemann GP Höher J Karamanidis K . Altered landing mechanics in ACL-reconstructed patients. Med Sci Sports Exerc. 2013;45(3):506-513. 23034645 10.1249/MSS.0b013e3182752ae3

[41] Pairot-de-Fontenay B Willy RW Elias ARC Mizner RL Dubé MO Roy JS . Running biomechanics in individuals with anterior cruciate ligament reconstruction: a systematic review. Sports Med. 2019;49(9):1411-1424. 31102111 10.1007/s40279-019-01120-x

[42] Patel AD Bullock GS Wrigley J Paterno MV Sell TC Losciale JM . Does sex affect second ACL injury risk? A systematic review with meta-analysis. Br J Sports Med. 2021;55(15):873. 34001504 10.1136/bjsports-2020-103408

[43] Paterno MV Ford KR Myer GD Heyl R Hewett TE . Limb asymmetries in landing and jumping 2 years following anterior cruciate ligament reconstruction. Clin J Sport Med. 2007;17(4):258-262. 17620778 10.1097/JSM.0b013e31804c77ea

[44] Paterno MV Rauh MJ Schmitt LC Ford KR Hewett TE . Incidence of second ACL injuries 2 years after primary ACL reconstruction and return to sport. Am J Sports Med. 2014;42(7):1567-1573. 24753238 10.1177/0363546514530088 PMC4205204

[45] Paterno MV Schmitt LC Ford KR , et al. Biomechanical measures during landing and postural stability predict second anterior cruciate ligament injury after anterior cruciate ligament reconstruction and return to sport. Am J Sports Med. 2010;38(10):1968-1978. 20702858 10.1177/0363546510376053 PMC4920967

[46] Petushek E Nilstad A Bahr R Krosshaug T . Drop jump? Single-leg squat? Not if you aim to predict anterior cruciate ligament injury from real-time clinical assessment: a prospective cohort study involving 880 elite female athletes. J Orthop Sports Phys Ther. 2021;51(7):372-378. 34192883 10.2519/jospt.2021.10170

[47] Pollard CD Stearns KM Hayes AT Heiderscheit BC . Altered lower extremity movement variability in female soccer players during side-step cutting after anterior cruciate ligament reconstruction. Am J Sports Med. 2015;43(2):460-465. 25512664 10.1177/0363546514560153

[48] Poston GR Schmitt LC Ithurburn MP Hugentobler JA Thomas S Paterno MV . Reduced 2-D frontal plane motion during single-limb landing is associated with risk of future anterior cruciate ligament graft rupture after anterior cruciate ligament reconstruction and return to sport: a pilot study. J Orthop Sports Phys Ther. 2021;51(2):82-87. 33356796 10.2519/jospt.2021.9302

[49] Ranganathan P Pramesh CS Aggarwal R . Common pitfalls in statistical analysis: logistic regression. Perspect Clin Res. 2017;8(3):148-151. 28828311 10.4103/picr.PICR_87_17 PMC5543767

[50] Schmitt LC Paterno MV Ford KR Myer GD Hewett TE . Strength asymmetry and landing mechanics at return to sport after anterior cruciate ligament reconstruction. Med Sci Sports Exerc. 2015;47(7):1426-1434. 25373481 10.1249/MSS.0000000000000560 PMC4418954

[51] Stearns KM Pollard CD . Abnormal frontal plane knee mechanics during sidestep cutting in female soccer athletes after anterior cruciate ligament reconstruction and return to sport. Am J Sports Med. 2013;41(4):918-923. 23425687 10.1177/0363546513476853

[52] Steffen K Nilstad A Kristianslund EK Myklebust G Bahr R Krosshaug T . Association between lower extremity muscle strength and noncontact ACL injuries. Med Sci Sports Exerc. 2016;48(11):2082-2089. 27327027 10.1249/MSS.0000000000001014

[53] Wiggins AJ Grandhi RK Schneider DK Stanfield D Webster KE Myer GD . Risk of secondary injury in younger athletes after anterior cruciate ligament reconstruction: a systematic review and meta-analysis. Am J Sports Med. 2016;44(7):1861-1876. 26772611 10.1177/0363546515621554 PMC5501245

