# Supplementary material for: Knee Biomechanics During Cutting Maneuvers and Secondary ACL Injury Risk: A Prospective Cohort Study of Knee Biomechanics in 756 Female Elite Handball and Soccer Players
Source: Am J Sports Med. 2024 Mar 8;52(5):1209–19. doi: 10.1177/03635465241234255 (PMC10986153; doi:10.1177/03635465241234255)
Supplement: sj-pdf-1-ajs-10.1177_03635465241234255 – Supplemental material for Knee Biomechanics During Cutting Maneuvers and Secondary ACL Injury Risk: A Prospective Cohort Study of Knee Biomechanics in 756 Female Elite Handball and Soccer Players [file sj-pdf-1-ajs-10.1177_03635465241234255.pdf]

Knee biomechanics during cutting maneuvers and secondary ACL injury risk – a combined prospective cohort and cross-sectional study of knee biomechanics in 756 female elite handball and football players

Appendix

Table A1. Comparison of participant characteristics, knee biomechanics and cutting technique variables between players with a secondary ipsilateral ACL injury (shaded grey) and the remaining three groups (n = 731 for kinematics; n = 690 for kinetics).

	Prev/New ACL group, ipsilateral leg of players with a new ipsilateral injury (n=6)	Prev ACL group, ipsilateral leg (n=49)	p	MD [95% CI]	New ACL group, leg with new injury (n=46)	p	MD [95% CI]	No ACL group, random leg (n=630)	p	MD [95% CI]
Age (y)	20.9 ± 2.1	23.3 ± 4.2	0.163	2.5 [-1.0, 5.9]	19.3 ± 3.1	0.367	1.6 [-1.9, 5.1]	20.7 ± 3.9	0.907	0.2 [-3.1, 3.5]
Body mass (kg)	74.8 ± 10.2	66.8 ± 7.2	**0.014**	**8.0* [1.6, 14.3]**	66.4 ± 7.6	**0.010**	**8.4* [2.0, 14.8]**	66.0 ± 7.7	**0.005**	**8.8* [2.7, 14.8]**
Height (cm)	176.3 ± 7.4	170.1 ± 6.0	**0.020**	**6.2* [1.0, 11.4]**	170.1 ± 7.2	**0.022**	**6.1* [0.9, 11.3]**	169.4 ± 6.2	**0.007**	**6.9* [1.9, 11.8]**
Time since injury (years)	1.6 ± 0.4	3.8 ± 2.6	0.050	2.2 [-0.0, 4.4]						
Knee flexion angle at IC (°)	28.7 ± 6.3	24.4 ± 8.5	0.132	4.3 [-1.3, 9.9]	22.9 ± 7.5	**0.043**	**5.8* [0.2, 11.4]**	24.3 ± 7.8	0.102	4.5 [-0.9, 9.8]
Knee flexion angle peak (°)	66.0 ± 6.0	62.0 ± 7.4	0.190	4.0 [-2.0, - 10.1]	61.1 ± 7.2	0.111	4.9 [-1.1, 11.0]	63.1 ± 6.7	0.317	2.9 [-2.8, 8.7]
Knee abduction angle at IC (°)	3.1 ± 4.3	3.6 ± 4.3	0.839	0.4 [-3.6, 4.4]	4.3 ± 4.2	0.568	1.2 [-2.8, 5.2]	5.0 ± 4.5	0.341	1.8 [-2.0, 5.6]
Knee abduction angle peak (°)	8.8 ± 4.6	9.3 ± 5.3	0.834	0.5 [-4.2, 5.2]	10.8 ± 4.3	0.405	2.0 [-2.7, 6.7]	10.9 ± 5.4	0.342	2.2 [-2.3, 6.7]
Knee abduction moment peak ($N \cdot m \cdot kg^{-1}$)	1.09 ± 0.26	1.39 ± 0.52	0.330	0.30 [-0.31 , 0.91]	1.82 ± 0.59	**0.018**	**0.74* [0.13, 1.35]**	1.68 ± 0.54	0.051	0.59 [-0.00, 1.18]
Knee internal rotation moment peak ($N \cdot m \cdot kg^{-1}$)	0.35 ± 0.37	0.18 ± 0.20	0.145	0.17 [-0.06 , 0.40]	0.24 ± 0.19	0.314	0.12 [-0.11, 0.35]	0.24 ± 0.20	0.294	0.11 [-0.10, 0.34]
Cutting angle (°)	83.5 ± 21.4	71.8 ± 18.9	0.218	11.7 [-6.9, 30.3]	65.4 ± 19.4	0.057	18.1 [-0.5, 36.7]	66.2 ± 21.0	0.055	17.3 [-0.4, 35.0]
Approach speed at IC ($m \cdot s^{-1}$)	2.98 ± 0.50	2.97 ± 0.42	0.979	0.01 [-0.35, 0.36]	3.27 ± 0.47	0.115	0.29 [-0.07, 0.65]	3.11 ± 0.50	0.461	0.13 [-0.21 , 0.47]

Values are means ± SD. ACL, anterior cruciate ligament; MD, mean difference; CI, confidence interval; IC, initial contact; Prev/New ACL group, players with a previous ACL injury who went on to sustain a new ipsilateral secondary ACL injury; Prev ACL group, players with a previous ACL injury only; New ACL group, players without a previous ACL injury who went on to sustain a new primary ACL injury; No ACL group, injury free players.
*significant mean difference (p≤ 0.05).

Table A2. Comparison of participant characteristics, knee biomechanics and cutting technique variables between players with a secondary contralateral ACL injury (shaded grey) and the remaining three groups (n = 734 for kinematics; n = 694 for kinetics).

	**Prev/New ACL group, contralateral leg of players with a new contralateral injury (n=8)**	**Prev ACL group, contralateral leg (n=50)**	**p**	**MD [95% CI]**	**New ACL group, leg with new injury (n=46)**	**p**	**MD [95% CI]**	**No ACL group, random leg (n=630)**	**p**	**MD [95% CI]**
Age (y)	24.2 ± 3.9	23.3 ± 4.2	0.566	0.9 [-2.1, 3.8]	19.3 ± 3.1	**0.001**	**4.9* [1.9, 7.9]**	20.7 ± 3.9	**0.013**	**3.5* [0.7, 6.3]**
Body mass (kg)	69.8 ± 7.4	66.8 ± 7.2	0.279	3.0 [-2.4, 8.4]	66.4 ± 7.6	0.217	3.4 [-2.0, 8.9]	66.0 ± 7.7	0.146	3.8 [-1.3, 8.9]
Height (cm)	170.1 ± 7.7	170.1 ± 6.0	0.989	0.0 [-4.4, 4.5]	170.1 ± 7.2	0.987	0.0 [-4.5, 4.5]	169.4 ± 6.2	0.737	0.7 [-3.5, 4.9]
Time since injury (years)	3.2 ± 1.9	3.8 ± 2.6	0.558	0.6 [-1.4, 2.5]						
Knee flexion angle at IC (°)	23.6 ± 11.5	24.9 ± 7.8	0.610	1.2 [-3.6, 6.0]	22.9 ± 7.5	0.773	0.7 [-4.1, 5.5]	24.3 ± 7.8	0.780	0.6 [-3.9, 5.1]
Knee flexion angle peak (°)	61.5 ± 6.2	63.6 ± 6.4	0.430	2.1 [-3.1, 7.2]	61.1 ± 7.2	0.865	0.4 [-4.7, 5.6]	63.1 ± 6.7	0.531	1.5 [-3.3, 6.4]
Knee abduction angle at IC (°)	6.2 ± 3.8	4.4 ± 4.0	0.299	1.8 [-1.6, 5.2]	4.3 ± 4.2	0.270	1.9 [-1.5, 5.4]	5.0 ± 4.5	0.443	1.2 [-1.9, 4.4]
Knee abduction angle peak (°)	11.5 ± 5.4	10.0 ± 4.9	0.467	1.5 [-2.5, 5.5]	10.8 ± 4.3	0.722	0.7 [-3.3, 4.8]	10.9 ± 5.4	0.769	0.6 [-3.2, 4.3]
Knee abduction moment peak (N·m·kg^{-1})	1.75 ± 0.32	1.57 ± 0.56	0.389	0.18 [-0.23, 0.60]	1.82 ± 0.59	0.729	0.07 [-0.35, 0.49]	1.68 ± 0.54	0.705	0.08 [-0.31, 0.46]
Knee internal rotation moment peak (N·m·kg^{-1})	0.24 ± 0.28	0.28 ± 0.24	0.662	0.04 [-0.12, 0.19]	0.24 ± 0.19	0.954	0.01 [-0.15, 0.16]	0.24 ± 0.20	0.941	0.01 [-0.14, 0.15]
Cutting angle (°)	72.3 ± 19.7	70.9 ± 20.4	0.868	1.4 [-14.7, 17.5]	65.4 ± 19.4	0.403	6.9 [-9.3, 23.0]	66.2 ± 21.0	0.428	6.1 [-8.9, 21.1]
Approach speed at IC (m·s^{-1})	2.92 ± 0.54	3.07 ± 0.45	0.323	0.16 [-0.15, 0.47]	3.27 ± 0.47	**0.028**	**0.35* [0.04, 0.66]**	3.11 ± 0.50	0.201	0.19 [-0.10, 0.48]

Values are means ± SD. ACL, anterior cruciate ligament; MD, mean difference; CI, confidence interval; IC, initial contact; Prev/New ACL group, players with a previous ACL injury who went on to sustain a new contralateral secondary ACL injury; Prev ACL group, players with a previous ACL injury only; New ACL group, players without a previous ACL injury who went on to sustain a new primary ACL injury; No ACL group, injury free players. *significant mean difference ($p \leq 0.05$).
